# Visceral Leishmaniasis and Immunocompromise as a Risk Factor for the Development of Visceral Leishmaniasis: A Changing Pattern at The Hospital for Tropical Diseases, London

**DOI:** 10.1371/journal.pone.0121418

**Published:** 2015-04-01

**Authors:** Kate Fletcher, Rita Issa, D. N. J. Lockwood

**Affiliations:** 1 Hospital for Tropical Diseases and London School of Hygiene & Tropical Medicine, London, United Kingdom; 2 The Hospital for Tropical Diseases, University college Foundation Trust, London, WC1E 6AU, United Kingdom; Royal Tropical Institute, NETHERLANDS

## Abstract

Visceral leishmaniasis (VL) is a parasitic protozoon infection caused by the *Leishmania* species and transmitted by sandflies. Patients acquire VL in five main tropical areas and the Mediterranean basin, and clinicians from non-endemic regions regularly see infected patients. We describe the population presenting with VL to the Hospital for Tropical Diseases (HTD), London and identify risk factors for developing VL.

**Methods and Principal Findings:**

A retrospective study of imported VL to the HTD, London including patients diagnosed and/or managed at the HTD between January 1995 and July 2013. We analyse patient demographics, risk factors for developing VL, diagnosis, investigation, management and outcome. Twenty-eight patients were treated for VL at the HTD over an 18 year period. The median age at VL diagnosis was 44 years (range 4–87 years) with a male to female ratio of 2:1. Most patients were British and acquired their infection in the Mediterranean basin. The median time from first symptom to diagnosis was six months with a range of 1–12 months and diagnosis included microscopic visualisation of leishmania amastigotes, positive serological tests (DAT and k39 antibody) or identification of leishmania DNA. Nineteen patients had some form of immunocompromise and this has increased proportionally compared to previously described data. Within the immunocompromised group, the ratio of those with autoimmune disease has increased. Immunocompromised patients had lower cure and higher relapse rates.

**Conclusions:**

The rise of VL in patients with immunocompromise secondary to autoimmune disease on immunomodulatory drugs presents new diagnostic and therapeutic challenges. VL should be a differential diagnosis in immunocompromised patients with pyrexia of unknown origin returning from travel in leishmania endemic areas.

## Introduction

Visceral leishmaniasis (VL) is a parasitic infection caused by the Leishmania species and is transmitted by the sandfly. [1] The annual global incidence of VL is over half a million cases in the endemic zones of Nepal, India, Bangladesh, Sudan, Brazil and the Mediterranean basin [2] With increasing global travel, clinicians from non-endemic regions are encountering more patients with VL infection.[3,4]

Patients with VL present with chronic pyrexia, anorexia, splenomegaly and pancytopenia. However there is a spectrum of clinical disease that depends upon the interplay between the host immune response and the parasite species and load. [5, 6]

In healthy, immunocompetent hosts, leishmania protozoa are killed by T- helper cells (Th), especially Th-1. Th-1 secrete several cytokines (IL-2, INF gamma and TNF alpha) recruiting and activating macrophages that phagocytose the cells with leishmania amastigotes.[7] In the immunosuppressed patients, T cell responses are inadequate and patients have increased susceptibility to developing clinical disease, experience a more severe disease course and have higher rates of relapse. [4, 8] HIV infection is an established risk factor for developing VL. [9,10]

In the UK patients with VL are treated at the HTD, London. Whilst risk factors for developing VL in endemic zones are well defined, [11] there is little published evidence of the risk factors for developing VL in a non-endemic setting.

We present a retrospective study of imported VL cases diagnosed and/or managed at the HTD, London between 1995 and 2013, and identify new risk factors for developing VL.

## Materials and Methods

Demographic and basic clinical details for all patients seen at the HTD are prospectively collected onto a database (Microsoft Access). Twenty-eight patients with VL were identified and the case notes and computerized records of these patients were reviewed. Using a standardised electronic proforma we recorded demographic data and information on past medical history, time to and method of diagnosis, treatment and outcomes.

The study was reviewed and approved by the Audit and Research Committee at the Hospital for Tropical Diseases, London who granted ethical approval for the study and stated that individual patient consent was not required as this was a retrospective case note review where the data was fully anonymised and de-identified prior to analysis.

### Case definitions

#### Visceral Leishmaniasis

Symptoms and signs suggestive of VL (chronic pyrexia, splenomegaly, pancytopenia) AND laboratory diagnosis of VL as defined below:

1. Visualisation of amastigotes in aspirated tissue material or biopsied tissue sections.[12]
2. A positive Direct Agglutination Test (DAT) which detects antibodies to leishmania protozoa.[12]
3. A positive rK39 rapid antibody test which detects antibodies to a protein-encoding gene (K39) found in leishmania species.[13,14]
4. Identification of Leishmania DNA using polymerase chain reaction techniques [12]

#### Immunocompromise

The authors discussed each patient and agreed that one or more of the domains below were satisfied. Four categories of immunocompromise were created and each patient was reviewed to see if they belonged to any category.

1. Condition known to cause immunocompromise
2. Treatment with immunosuppressive drugs
3. Immunocompromising co-morbidity
4. Lifestyle known to cause immunocompromise

#### Cure

Completion of treatment course AND resolution of clinical signs and symptoms AND improvement or normalisation of laboratory parameters

#### Relapse

Achieved cure as defined above AND development of new episode of VL as defined above

## Results

There were 28 patients with VL, the male to female ratio was 2:1 and median age at diagnosis 44 years. Two thirds were British in origin, with the remainder from Algeria, Cyprus, Eritrea, Ethiopia, India, Italy and Spain.

**Fig 1:**
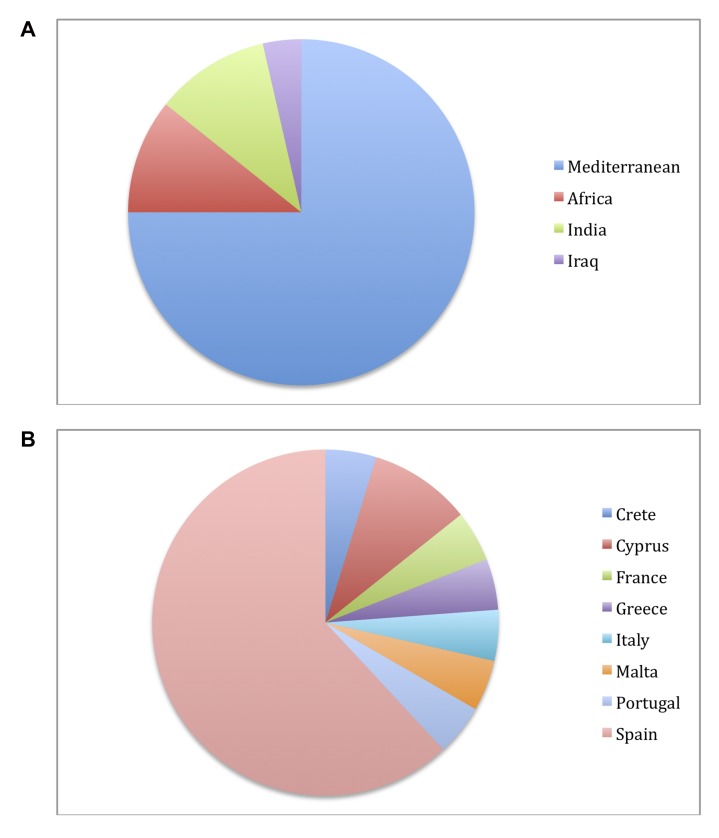
a: Areas of Acquisition for whole cohort. b: Countries of Acquisition in Mediterranean Basin for whole cohort.

Patients holidayed in an endemic region (16 patients). Three patients lived in endemic zones and subsequently emigrated, two patients were visiting friends and relatives (VFR) and two patients were working. See below for (Fig 1) more detail on areas of acquisition of VL.

Nine patients were identified as immunocompetent and nineteen as immunocompromised. The data for these two categories will be presented separately.

### Immunocompetent Patients

Median age at diagnosis was 38 and the male to female ratio was 2:1. All but one of the patients (1 Cypriot) were of British origin. Six of the nine patients had holidayed in the endemic regions, and three worked or had a holiday home there. The median time to diagnosis was six months, range of 3–12 months.

Leishmania amastigotes were seen in biopsies taken from all immunocompetent patients (six bone marrow aspirates, one subcutaneous nodule, one splenic biopsy and one splenic aspirate). Serology was performed on seven of the patients, all of whom were positive on DAT and k39 antigen. In three patients, the causative species of protozoa was identified by DNA PCR as Leishmania donovani.

All patients received liposomal amphotericin B. Eight of the nine immunocompetent patients were cured. One patient developed mucocutaneous leishmaniasis (ML)13 years later (VL diagnosis 1996, ML diagnosis 2009) requiring treatment with sodium stibogluconate and was then cured.

### Immunocompromised Patients

There were 19 patients in the immunocompromised category. Seven patients were HIV positive and five were on highly active anti-retroviral therapy (HAART) when their VL was diagnosed. See Table 1 for a summary of these patients.

**Table 1:** Patients with retroviral disease.

	Therapy and markers at time of VL diagnosis	ARV Alteration	Relapse
**1**	**CD4 count = 269 cells/ mm** ^**3**^ Saquinavir 1g BD Ritonavir 100mg BD Tenofovir 245mg OD Namivudine 150mg BD	ARV changed by HIV team to Saquinavir, Ritonavir, Truvada (emtricitabine/ tenofovir).	VL relapsed: treated with ambisome, miltefosine and pentamidine prophylaxis.
**2**	**CD4 count = 143 cells/ mm** ^**3**^ **VL <40/ml** Darinuvir 800mg OD Ritonavir 100mg OD	No change in ARV.	VL relapsed: treated with ambisone.
**3**	**CD4 count = 140 cells/ mm** ^**3**^ **VL 80/ml** Kivexa 1 OD (abacavir/lamivudine) Kaletra 2 BD (Lopinavir/ritonavir) Patient also has steroid induced diabetes; HbA1c = 9	No change in ARV.	VL relapsed: successfully treated with ambisone.
**4**	**CD4 count = 120 cells/ mm** ^**3**^ **VL 40/ml** Atripla (efavirenz/emtricitabine/ tenofovir) Ritonavir	ARV’s changed due to rising viral load and nevirapine resistance. Started Darunavir and Truvada.	No VL relapse.
**5**	**CD4 count = 410 cells/ mm** ^**3**^ **VL 700 000/ml** Kaletra 2 BD	No change in ARV.	No VL relapse.
**6**	**CD4 count = not available**	Not on ARVs	No VL relapse
**7**	**CD4 count = not available**	Not on ARVs	Relapsed with Post Kala azar dermal Leishmaniasis Discharged

HIV-1 protease inhibitors (PI) are suggested for inclusion in patients co-infected with HIV and VL. Note all patients on ARV’s were already on Ritonavir (PI) at time of VL diagnosis.

Six patients had autoimmune disease, all of whom were taking immunomodulatory drugs at the time of diagnosis. Two further patients had haematological malignancies: chronic lymphoid leukaemia and T cell lymphoma. Of the remaining patients, their immunocompromise may have been attributable to diabetes, chronic alcohol excess or multiple co-morbidites. See Table 2 and S1 Fig for further information on these patients.

**Table 2:** Patients with Immunocompromise not attributable to retroviral disease.

	Diagnosis	Therapy	Relapse
**1**	Graves disease, autoimmune haemolytic anaemia, diabetes mellitus type 2 (HbA1c unknown).	Prednisolone 10mg OD Azathioprine 50mg OD Metformin (dose unknown)	No
**2**	Systemic lupus erythematosus	Prednisolone 6mg OD Hydroxychloroquine 400mg BD	No
**3**	Rheumatoid arthritis	Methotrexate (dose unknown). Stopped at time of VL diagnosis	No
**4**	Rheumatoid arthritis	Methotrexate (dose unknown) Steroids (unknown)	No
**5**	Psoriatic arthritis	Methotrexate (dose unknown) TNF-alpha inhibitor (dose unknown)—stopped when patient acutely unwell.	Yes
**6**	Psoriasis, CD4 lymphopaenia Developed ocular leishmaniasis.	Prednisolone 10mg OD Dapsone 50mg OD	Yes, patient still receiving pentamidine.
**7**	Diabetes mellitus type 2 (HbA1c unknown), alcohol induced pancreatitis (alcohol intake unknown).	Gliclazide and Metformin (doses unknown).	No
**8**	Diabetes mellitus type 2, cervical carcinoma, chronic kidney disease, ischemic heart disease, CD4 lymphopaenia	Polypharmacy—no immunomodulatory treatment.	Yes, discharged after cure
**9**	Chronic lymphoid leukaemia	Unknown medications.	No
**10**	Unspecified T cell lymphoma	Prednisolone 10mg OD.	Yes
**11**	Alcohol excess—80 units per week.		No
**12**	Alcohol excess—252 units per week, basal cell carcinoma, CD4 lymphopaenia		Yes, **o**n prophylaxis.

11 patients in the immunocompromised group were British. The remaining eight were from Spain, Italy, India, Algeria, Ethiopia and Eritrea. The country of acquisition of VL was predominantly in the Mediterranean basin, whilst three were in Africa and two in India. Ten patients holidayed in the endemic zone, four patients had a holiday home, two were visiting friends and relatives, two had migrated to the endemic zone and one patient was there to work. Median age at diagnosis was 48.5 years, male: female ratio was 2:1. The median time to diagnosis was five months with a range of 1–120 months.

Fourteen patients had Leishmania amastigotes identified on microscopy, twelve in bone marrow aspirates and two in splenic aspirates. Eight types of tissues were examined and Leishmania amastigotes found (four bone marrow, three duodenal, two gastric, two skin, one colon, one spleen, one lymph node and one liver). One patient was diagnosed on serological testing. L. donovani was identified as the infecting species by DNA PCR in15 patients from tissue samples. For a summary of the diagnostic investigations please refer to Table 3.

**Table 3:** Outcomes of diagnostic tests per immune status (Positive result/total number of test performed).

	Microscopy	Histology	Serology	DNA PCR	Culture
Immunocompetent	3/3	6/6	7/7	3/5	0/3
Immunocompromised	14/16	9/9	10/11	15/18	3/11

Most patients (n = 18) were treated with liposomal amphotericin B. Nine patients relapsed and needed further treatment. Of these nine patients, six were then treated with intermittent prophylactic medication (five pentamidine, one liposomal amphotericin B).

Of the nine patients who relapsed the immunocompromised subgroups were as follows: four patients with HIV infection, two patients with psoriatic arthropathy, one patient with multiple co mobidities including diabetes mellitus, one with a T cell lymphoma and one with a history of excess alcohol.

Three patients were subsequently found to have a CD4 lymphompenia. See Table 2.

Nine of the 19 were cured and discharged (no longer seen at the HTD for their VL > 1 year).

Four patients died during the study period. One died from complications of a VL relapse which resulted in sepsis and ARDS. One patient died from chronic lymphoid leukaemia and for the remaining two patients the cause of death was not available. Six remain under the care of the HTD either on VL prophylaxis or for continued surveillance of their VL. For a summary of results for the whole cohort please refer to Table 4.

**Table 4:** Summary of Results.

	Immunocompetent patients (n = 9)	Immunocompromised patients (n = 19)
**Demographics**	Median age at diagnosis = 38 M:F = 2:1. 8 British, 1 Cypriot	Median age at diagnosis = 48.5 years M:F = 2:1 11 British, 1 Spanish, 1 Italian, 1 Indian, 1 Algerian, 1 Ethiopian, and 1 Eritrean. (2 patients country of origin not recorded)
**Reason in endemic zone**	6 Holiday 2 holiday home 1 work	10 Holiday; 4 holiday home; 2 visiting friends and relatives; 2 migrated to the endemic zone; 1 for work
**Median time to diagnosis**	6 months, range of 3–12 months	5 months, range of 1–120 months
**Diagnostic method**	Microscopy: 6 bone marrow biopsy, 1 subcutaneous nodule, 1 splenic biopsy, 1 splenic aspirate. Serology:7 positive DAT and k39 antigen. DNA PCR: L. donovani identified in 3 patients	Microscopy: 12 in bone marrow aspirates, 2 in splenic aspirates, 8 types of tissues were examined and Leishmania amastigotes found: bone marrow 4, duodenal 3, gastric 2, skin 2, colon 1, spleen 1, lymph node 1 liver. Serology:1 patient was diagnosed on serological testing. DNA PCR: L. donovani identified in 15 patients
**Treatment**	All received liposomal amphotericin B	18 treated with liposomal amphotericin B. 9 patients relapsed requiring further treatment 6 patients needed intermittent prophylactic Medication. **Pentamidine Prophylaxis** Patient 1:3 weekly for 2 years, Patient 2:2–3 weekly for >6 years, Patient 3:Monthly recorded for 1 year, Patient 4:Monthly, unknown duration, Patient 5:3 weekly recorded for 1 year, **Liposomal amphotericin B Prophylaxis**, Patient 6: monthly
**Outcome**	8 of the 9 were cured and discharged. 1 later developed mucocutaneous leishmaniasis, was treated with sodium stibogluconate and was then cured.No deaths recorded.	9 of the 19 were cured and discharged. 4 died during the study period. 1 patient died from complications of a VL relapse, which resulted in sepsis and ARDS. 1 patient died from chronic lymphoid leukaemia and for the remaining 2 patients the cause of death was not available. 6 remain under the care of the HTD on VL prophylaxis or for continued surveillance of their VL.

## Discussion

The strengths of this study are the complete data set which is a representative cohort from the HTD. Retrospective data collection and being unable to capture all patients in the UK with VL during this time period are limitations.

Up to two patients with VL are seen at the HTD annually.[3] The majority of patients are British tourists travelling to the Mediterranean basin. Our new finding is that patients with medical immunosuppression are now a significant group at risk of developing VL.

Two thirds of the cohort examined had some form of immunocompromise. HIV positive patients comprise the largest subgroup of immunocompromised patients at 41% of the cohort, followed by patients with autoimmune diseases (35%). This is the first report of an association between VL and autoimmune diseases in a cohort. We found a change in the type of patients who are developing VL compared to previous publications. [4] The proportion of patients with VL and immunocompromise has increased with the biggest increase in the autoimmune disease group. The decline in the proportion with HIV may be due to the widespread use of highly active anti-retroviral therapy. [15] The increase in patients with autoimmune diseases and VL may result from more patients with chronic autoimmune diseases being able to tolerate immunomodulation and to travel to endemic zones. [16,17]

The two groups had a similar median time to diagnosis however the absolute ranges were very different. Further to this the median age at diagnosis in the immunocompromised group was over ten years older than the immunocompetent group (38 years, 48.5 years). These findings reflect the diagnostic difficulty posed by VL in the immunocompromised patient whose presentations can be atypical.

Of the nine immunocompromised patients who relapsed, three were found to have a CD4 lymphopenia. All of these patients remained lymphopenic after treatment and suffered relapses. Two patients developed disseminated disease where amastigotes were found in atypical locations such as the gastrointestinal tract and in ocular tissues. No other patients in the cohort developed new lymphopenia after treatment for VL.

The patients with CD4 lymphopenia were identified at the time of their VL diagnosis. We have no record of their lymphocyte counts prior to this and therefore are unable say if the low lymphocyte count was the risk factor for developing VL or a consequence of the disease itself. However despite treatment and cure in one of these patients their lymphocyte counts have remained low which could suggest that the lymphopenia predated the VL. The final two patients remain on VL prophylaxis.

We know that diabetes and chronic alcohol excess can impair the immune system by impacting on T lymphocyte function [18,19]. Despite limited evidence of the link between these risk factors and the development of VL, because T cells are critical in controlling leishmania protozoa, an absolute reduction in lymphocyte number as well as impaired function of the remaining lymphocytes could contribute to the development and relapse of VL. For example, within our cohort, two patients with CD4 lymphopenia and either diabetes or alcohol excess developed VL relapses. However, the small patient numbers mean that diabetes and alcohol excess remain only potential risk factors for the development of a severe VL course.

We would recommend that a differential lymphocyte count with CD4 cell subsets be included in the routine work up for patients who are diagnosed with VL.

We would also suggest raising awareness of VL within the rheumatological community and that VL be considered as part of the differential diagnosis in rheumatology patients with pyrexia of unknown origin who return from travel to endemic areas.

## Supporting Information

S1 Fig Timeline of VL Diagnosis in Immunocompromised Patients. (TIFF)
