# Supplementary figures and images for: Visceral Leishmaniasis and Immunocompromise as a Risk Factor for the Development of Visceral Leishmaniasis: A Changing Pattern at The Hospital for Tropical Diseases, London
Source: PLoS One. 2015 Apr 1;10(4):e0121418. doi: 10.1371/journal.pone.0121418 (PMC4382278; doi:10.1371/journal.pone.0121418)

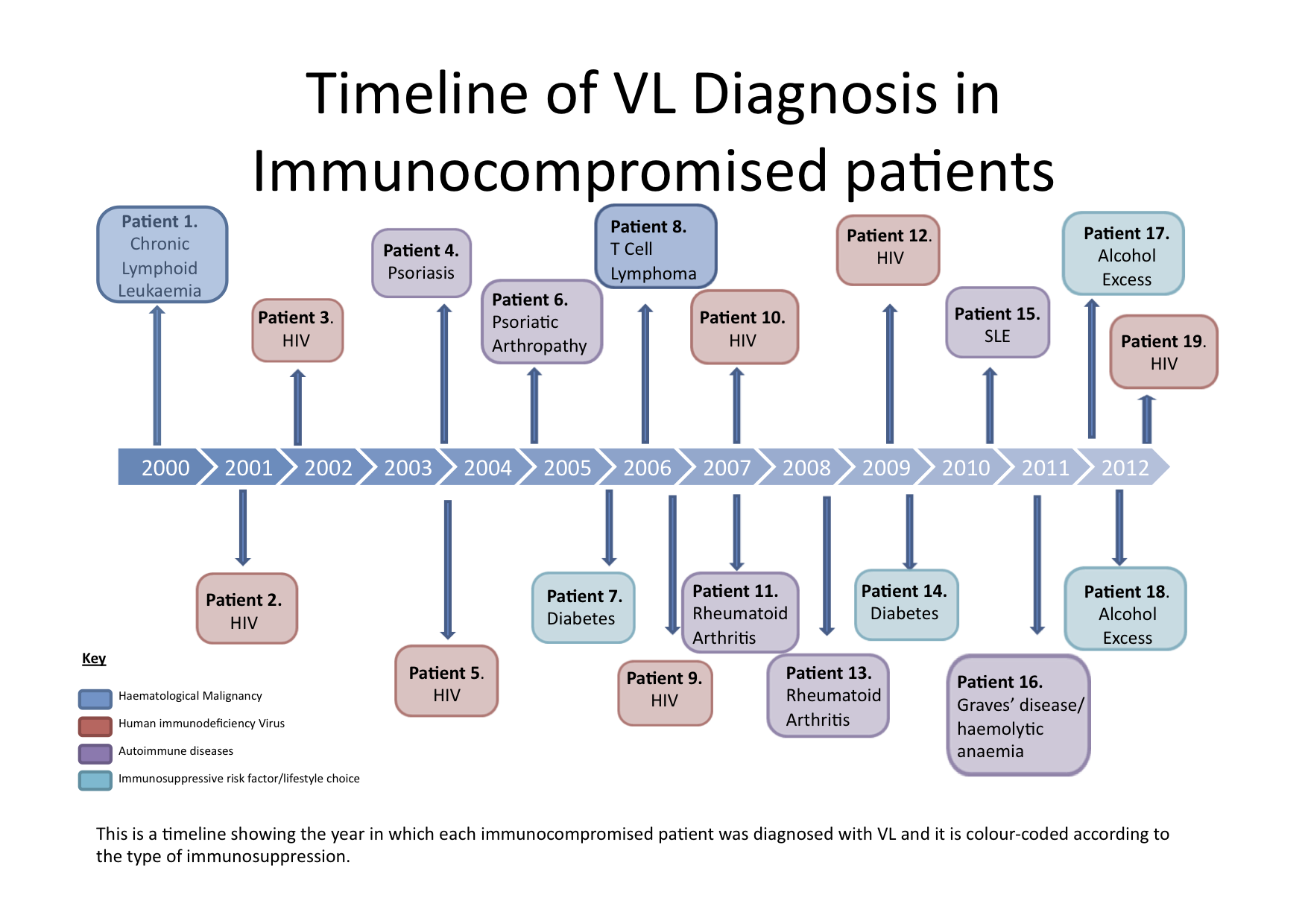

Supplement: S1 Fig — (TIFF) [file pone.0121418.s001.tiff]
